# Computer simulation allows goal-oriented mechanical ventilation in acute respiratory distress syndrome

**DOI:** 10.1186/cc5719

**Published:** 2007-03-12

**Authors:** Leif Uttman, Helena Ögren, Lisbet Niklason, Björn Drefeldt, Björn Jonson

**Affiliations:** 1 Department of Clinical Physiology, Lund University, 221 85 Lund, Sweden

## Abstract

**Introduction:**

To prevent further lung damage in patients with acute respiratory distress syndrome (ARDS), it is important to avoid overdistension and cyclic opening and closing of atelectatic alveoli. Previous studies have demonstrated protective effects of using low tidal volume (V_T_), moderate positive end-expiratory pressure and low airway pressure. Aspiration of dead space (ASPIDS) allows a reduction in V_T _by eliminating dead space in the tracheal tube and tubing. We hypothesized that, by applying goal-orientated ventilation based on iterative computer simulation, V_T _can be reduced at high respiratory rate and much further reduced during ASPIDS without compromising gas exchange or causing high airway pressure.

**Methods:**

ARDS was induced in eight pigs by surfactant perturbation and ventilator-induced lung injury. Ventilator resetting guided by computer simulation was then performed, aiming at minimal V_T_, plateau pressure 30 cmH_2_O and isocapnia, first by only increasing respiratory rate and then by using ASPIDS as well.

**Results:**

V_T _decreased from 7.2 ± 0.5 ml/kg to 6.6 ± 0.5 ml/kg as respiratory rate increased from 40 to 64 ± 6 breaths/min, and to 4.0 ± 0.4 ml/kg when ASPIDS was used at 80 ± 6 breaths/min. Measured values of arterial carbon dioxide tension were close to predicted values. Without ASPIDS, total positive end-expiratory pressure and plateau pressure were slightly higher than predicted, and with ASPIDS they were lower than predicted.

**Conclusion:**

In principle, computer simulation may be used in goal-oriented ventilation in ARDS. Further studies are needed to investigate potential benefits and limitations over extended study periods.

## Introduction

In patients with acute lung injury and acute respiratory distress syndrome (ARDS), adequate gas exchange requires mechanical ventilation; however, this can aggravate the condition by causing ventilator-induced lung injury (VILI), particularly at high tidal volume (V_T_) and high airway pressure [1-4]. Tidal lung collapse and re-expansion causing shear forces should be avoided [5]. Lung-protective ventilation may be based on low V_T_, low postinspiratory plateau pressure (P_PLAT_) and adequate positive end-expiratory pressure (PEEP) [2-4,6]. An adequate PEEP should reduce alveolar collapse occurring at the end of expiration. A low V_T _prevents high P_PLAT _and alveolar overdistension [7,8]. Permissive hypercapnia, partial liquid ventilation, extracorporeal carbon dioxide removal, high-frequency oscillatory ventilation, tracheal gas insufflation and aspiration of dead space (ASPIDS) are strategies that have been developed to prevent VILI [9-13].

When ASPIDS is used during late expiration, a defined volume of gas (ASPIDS volume) is aspirated through a catheter from the tip of the tracheal tube and simultaneously replaced with fresh gas through the ordinary lumen of the tracheal tube. ASPIDS increases carbon dioxide removal by reducing dead space. De Robertis and coworkers [13-15] showed that ASPIDS allows decreased V_T _and airway pressures in healthy animals as well as in ARDS patients. Liu and colleagues [16] used ASPIDS to lower V_T _in patients with exacerbation of chronic obstructive pulmonary disease, resulting in reduced airway pressures and arterial carbon dioxide tension (Paco_2_). The potential of ASPIDS to decrease V_T _is not studied at high respiratory rates (RRs).

A critically ill patient connected to a ventilator represent a very complex system. It is virtually impossible, even for experienced physicians, to know the best combination of, for instance, V_T_, PEEP, RR and inspiratory/expiratory ratio that leads to specific physiological goals. Such goals may be to maintain an adequate Paco_2 _and a limited P_PLAT _at minimal V_T_.

A tool for decision support when resetting the ventilator was previously developed [17,18]. In principle, that tool is based on the patient's physiological lung function profile, enabling computer simulation of mechanics and gas exchange at alternative ventilator settings. Hence, the consequences of an intended adjustment in ventilator settings may be analyzed in advance. In theory, that tool may be used iteratively in order to identify the optimal ventilator settings that will lead to predefined goals.

We tested the hypothesis that, by applying goal-orientated ventilation based on iterative computer simulation, V_T _can be reduced at high RR and much further reduced during ASPIDS, without compromising gas exchange or causing high airway pressure.

## Materials and methods

### Material and preparation

The local ethics board of animal research approved the study. Eight pigs of the Swedish native breed weighing 18 to 22 kg were used. The animals were pre-medicated with xylazine (2 mg/kg) and anaesthetized with ketamine (15 mg/kg). During the experiment anaesthesia was maintained by the continuous intravenous infusion of fentanyl (60 μg/kg·per hour) and midazolam (0.7 mg/kg·per hour). Initially the animals were hydrated with 1,000 ml ringer acetate, followed by infusion at 100 ml/hour. Ten millilitres of dextran 1 was infused, followed by 1,000 ml dextran 70 to avoid falling blood pressure. A catheter in the left femoral artery allowed monitoring of heart rate and arterial blood pressure, and sampling for immediate blood gas analysis (Radiometer ABL725, Copenhagen, Denmark). Body temperature was maintained constant. The animals were intubated and ventilated (Servo Ventilator 900C; Siemens-Elema AB, Solna, Sweden). Carbon dioxide concentration at the airway opening was measured using a mainstream carbon dioxide analyser (CO_2 _Analyzer 930; Siemens-Elema AB). The ventilator/computer system used to record data and control the ventilator has been described elsewhere [19].

### Protocol

Basal ventilation was volume-controlled square inspiratory flow pattern at 20 breaths/min, with inspiratory time at 33% of the respiratory cycle, post-inspiratory pause time at 5% and PEEP at 8 cmH_2_O. Fractional inspired oxygen Fio_2 _was 1.0.

#### ARDS induction

Surfactant perturbation was provoked by administration of the detergent dioctyl sodium sulphosuccinate in 5% aerosol form for 200 breaths, as previously described [20]. Pressure-controlled harmful ventilation was started with a P_PLAT _of 50 cmH_2_O and end-expiratory pressure of -10 cmH_2_O at 10 breaths/min. Dead space was added to maintain normocapnia. Harmful ventilation was continued for 90 min or until compliance (V_T_/[P_PLAT _- PEEP]) decreased by 25%. Harmful ventilation was stopped when substantial exudates appeared in the tracheal tube. ARDS was diagnosed if arterial oxygen tension (Pao_2_)/Fio_2 _was less than 27 kPa after 5 min at basal ventilation at PEEP 0 cmH_2_O. If this criterion was not met, harmful ventilation continued for another 30 min.

Ventilation was continued at 40 breaths/min and inspiratory:expiratory ratio 1:1 (inspiratory time 30% + postinspiratory pause time 20%) while PEEP was slowly increased to 15 cmH_2_O. Minute volume was adjusted to reach a Paco_2 _of 6.0 kPa. Sixty minutes was allowed for stabilization.

### Defining the physiological profile and computer simulation of resetting

**Figure 1:**
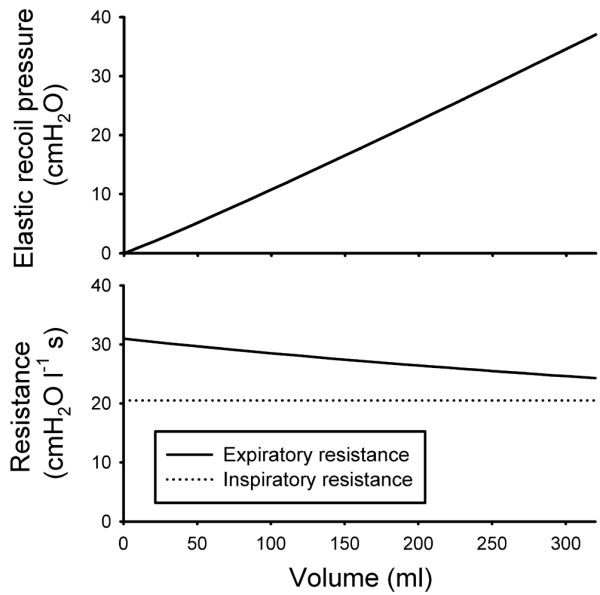
The physiologic profile with regard to mechanics. Elastic recoil pressure (upper) and resistance (lower) as a function of volume above end-expiratory volume at preset total positive end-expiratory pressure.

**Figure 2:**
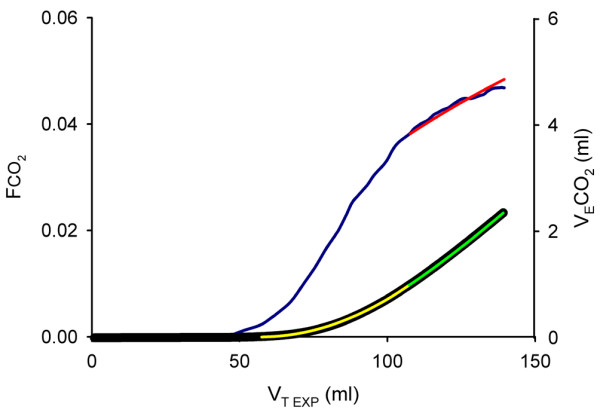
The physiologic profile with regard to carbon dioxide elimination. The blue curve represents carbon dioxide concentration at airway opening (Fco_2_) versus expired tidal volume (V_T EXP_). Its upper part was approximated by a logarithmic equation (red curve). Measured expired volume of carbon dioxide (V_E_co_2_) versus V_T EXP _(black curve) was obtained by integration of the blue curve. To describe V_E_co_2 _versus V_T EXP _mathematically, the lower part was expressed as a second-degree polynomial (yellow curve). At high V_T EXP _the logarithmic equation was integrated (green curve) to allow estimation of V_E_co_2_. This mathematical description of V_E_co_2 _versus V_T EXP _allows prediction of expired carbon dioxide volumes at an alternative tidal volume.

Signals from the ventilator and carbon dioxide analyzer representing flow rate, airway pressure, and carbon dioxide concentration at the airway opening were sampled using a personal computer at frequency of 100 Hz and transferred to a spreadsheet to derive a physiological profile, which is the basis of the computer simulation [17]. In short, the physiological profile consists of 17 parameters describing the elastic pressure/volume curve of the respiratory system, inspiratory and expiratory resistance as a function of volume, and how the volume of eliminated carbon dioxide varies with V_T _(Figures 1 and 2). A nonlinear elastic pressure/volume curve was constructed, using the following equation: elastic pressure = a·V^b^+PEEP_TOT_, where PEEP_TOT _is current total PEEP, read during a 0.5 s postexpiratory pause while all ventilator valves are closed and no flow exists.

Expired volume of CO_2 _(V_E_co_2_) was described in relation to expired V_T _(Figure 2) [17]. This gives a first estimate of V_E_co_2 _at a new V_T_. Carbon dioxide elimination is also dependent on time for gas distribution from the alveolar capillaries up to the fresh gas interface (mean distribution time) [21,22]. Hence, the change in mean distribution time at a new ventilator setting was simulated to obtain a second estimate of V_E_co_2 _using data from Uttman and Jonson [21]. The volume of carbon dioxide in the Y-piece and adjacent tubing re-inspired during early inspiration (V_I_co_2_) was calculated [17]. Then, tidal CO_2 _elimination (V_T_co_2_) was determined: V_T_co_2 _= V_E_co_2_-V_I_co_2_.

At steady state and at stable metabolic rate, Paco_2 _is related to the effective alveolar ventilation [23,24]. Accordingly, Paco_2 _at alternative ventilation was calculated from the effect on RR multiplied byV_T_co_2_:

PaCO2 PRED=PaCO2⋅RR⋅VTCO2RRALT⋅VTCO2 ALT
 MathType@MTEF@5@5@+=feaafiart1ev1aaatCvAUfKttLearuWrP9MDH5MBPbIqV92AaeXatLxBI9gBaebbnrfifHhDYfgasaacH8akY=wiFfYdH8Gipec8Eeeu0xXdbba9frFj0=OqFfea0dXdd9vqai=hGuQ8kuc9pgc9s8qqaq=dirpe0xb9q8qiLsFr0=vr0=vr0dc8meaabaqaciaacaGaaeqabaqabeGadaaakeaacqqGqbaucqqGHbqycqqGdbWqcqqGpbWtdaWgaaWcbaGaeGOmaiJaeeiiaaIaeeiuaaLaeeOuaiLaeeyrauKaeeiraqeabeaakiabg2da9iabbcfaqjabbggaHjabboeadjabb+eapnaaBaaaleaacqaIYaGmaeqaaOGaeyyXIC9aaSaaaeaacqqGsbGucqqGsbGucqGHflY1cqqGwbGvdaWgaaWcbaGaeeivaqfabeaakiabboeadjabb+eapnaaBaaaleaacqaIYaGmaeqaaaGcbaGaeeOuaiLaeeOuai1aaSbaaSqaaiabbgeabjabbYeamjabbsfaubqabaGccqGHflY1cqqGwbGvdaWgaaWcbaGaeeivaqfabeaakiabboeadjabb+eapnaaBaaaleaacqaIYaGmcqqGGaaicqqGbbqqcqqGmbatcqqGubavaeqaaaaaaaa@5D9E@

Where PaCO_2 PRED _is predicted Paco_2 _at alternative respiratory rate (RR_ALT_) and V_T_co_2 _(V_T_co_2 ALT_).

#### Goal-orientated ventilation

The principle underlying goal-orientated ventilation based on computer simulation has been described elsewhere [25,26]. In short, the operator defines the immediate physiological goals that should be achieved by ventilation. Starting from these goals and the physiological profile, the computer iteratively seeks the ventilator setting that is optimal for reaching these goals. In the present context, the simulation goals were defined with the intention being to minimize V_T_, maintain normocapnia (Paco_2 _= 6.0 kPa), avoid hypoxia (PEEP_TOT _≥ 10 cmH_2_O) and avoid overdistension (P_PLAT _= 30 cmH_2_O).

#### Minimal V_T _at high respiratory rate and at ASPIDS

Simulation was carried out by increasing RR in steps of 5 breaths/min, whereupon the computer iteratively adjusted V_T _and PEEP so as to achieve the goals. At simulation of each change to the ventilator settings, the following constraints were applied. To avoid the change leading to a nonsignificant reduction in V_T_, the V_T _should decrease by at least 0.25 ml for each unit increment in RR. To limit consequences of errors in determination of expiratory resistance, intrinsic PEEP should not be more than 50% of PEEP_TOT_. RR was allowed to increase by 20 breaths/min. The iterative simulation used the tool SOLVER in Excel 2003 (Microsoft, Redmond, WA, USA).

The ventilator was reset to achieve the V_T_, PEEP and RR indicated by the simulation. After a stabilization period of 30 minutes, a new physiological profile was determined and followed by another simulation. If the latter indicated a significant reduction in V_T_, then the ventilator settings were changed and the procedure repeated. The lowest V_T _achieved without ASPIDS was associated with a high respiratory rate (RR_HIGH_). Then, ASPIDS was simulated using the same constraints as above. The ventilator was reset and ASPIDS was activated during the last 50% of the expiratory time, using an ASPIDS volume of 0.5 V_T_+25 ml. A physiological profile was established after 30 and 60 min. The volume of gas insufflated in the inspiratory line during the ASPIDS period is a few millilitres higher than the volume of gas simultaneously aspirated from the tip of the tracheal tube. The PEEP regulation of the ventilator efficiently allows the slight surplus of gas to escape without affecting airway pressures. PEEP_TOT _was measured.

The animals were killed by an overdose of potassium chloride.

### Statistical methods

All data are expressed as mean ± standard deviation. We used Wilcoxon signed rank test to detect differences in respiratory parameters at different ventilator settings and to determine the accuracy of simulation.

## Results

### ARDS model

The ARDS criterion was met after 54 ± 29 min of harmful ventilation. Pao_2_/Fio_2 _was 14 ± 6.2 kPa at PEEP 0 cmH_2_O. Physiological dead space was 78 ± 4%.

### Computer simulation

**Figure 3:**
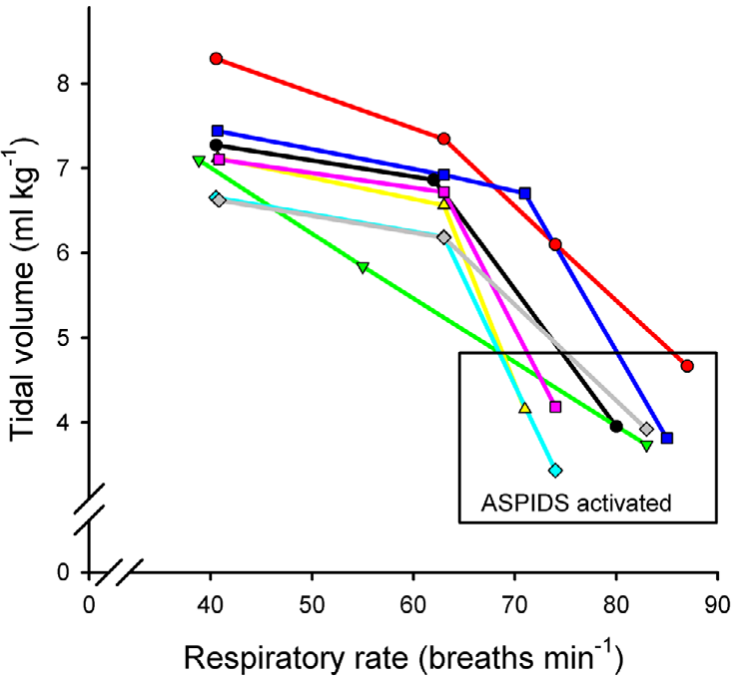
Tidal volume versus respiratory rate. Aspiration of dead space (ASPIDS) allowed an important reduction in tidal volume in all animals.

Under the guidance of simulation, the RR was increased from 40 to 64 ± 6 breaths/min as V_T _was reduced from 7.2 ± 0.5 to 6.6 ± 0.5 ml/kg (*P *= 0.005; Figure 3). At ASPIDS V_T _could, while remaining in accordance with the simulation, be reduced to 4.0 ± 0.4 ml/kg at RR of 80 ± 6 breaths/min (*P *= 0.005). The values for P_PLAT_, PEEP_TOT _and PaCO_2 _measured after ventilator resetting to RR_HIGH _and after resetting to ASPIDS for 30 min and 60 min were close to those predicted by the simulation (Table 1). However, P_PLAT _and PEEP_TOT _were higher than predicted at RR_HIGH _(*P *< 0.05) and lower than predicted at ASPIDS (*P *= 0.01). The fraction of re-inspired carbon dioxide (V_I_co_2_/V_E_co_2_) increased from 0.29 ± 0.02 at 40 breaths/min to 0.38 ± 0.02 at RR_HIGH _(*P *= 0.005). Pao_2 _was 66 ± 10 at 40 breaths/min and PEEP 15 cmH_2_O, and did not change at RR_HIGH _or at ASPIDS (*P *> 0.05).

**Table 1:** Simulated and measured values at minimal tidal volume

Parameter	RR_HIGH_		ASPIDS		
	
	Simulated	Measured	Simulated	Measured at 30 min	Measured at 60 min
P_PLAT _(cmH_2_O)	30	32 ± 2.2*	30	26 ± 1.5**	26 ± 1.4**
PEEP_TOT _(cmH_2_O)	17 ± 1.8	18 ± 1.7*	21 ± 1.7	17 ± 2.1**	17 ± 2.2**
Paco_2 _(kPa)	6.0	5.7 ± 0.70 NS	6.0	5.8 ± 0.42 NS	5.7 ± 0.68 NS

Postinspiratory plateau pressure (P_PLAT_), total positive end-expiratory pressure (PEEP_TOT_) and arterial carbon dioxide tension (Paco_2_) at lowest tidal volume found in simulation by increasing respiratory rate to high levels (RR_HIGH_) and by using aspiration of dead space (ASPIDS) for 30 and 60 min. Values are expressed as means ± standard deviation. Differences between measured and simulated values: *P < 0.05, **P = 0.01. NS, not significant.

## Discussion

In accordance with the hypothesis, V_T _was reduced at higher RRs but also, and most importantly, when ASPIDS was used. Computer simulation allowed titration of V_T _as RR was increased and when ASPIDS was used, with satisfactory achievement of goals in terms of airway pressure and gas exchange.

Because this study has its focus on technical and conceptual development, it has several limitations. This porcine ARDS model simulates clinical ARDS with respect to hypoxia. The physiological dead space is increased, as in patients [27,28]. Bitzén and coworkers [29] showed that the elastic pressure-volume diagrams demonstrated lung collapse and recruitment, as occur in patients with early ARDS. However, the model may differ from ARDS in patients with sepsis, obstruction and airway secretions. Moreover, in contrast to humans, pigs do not have collateral ventilation that equilibrates ventilatory heterogeneity [30].

Particularly at high RR, gas exchange may therefore differ between the porcine model and clinical ARDS. The concept behind the present study is that a combination of predefined immediate physiological goals should be met by selecting a mode of ventilator operation. Therefore, measurements were limited to 30 and 60 min after resetting. This time should be long enough for establishment of a new steady state, particularly with respect to Paco_2 _but not so long that important changes in physiological status of the animal would occur. In a tentative clinical setting, one would need to update the physiological profile at intervals reflecting the stability – or the instability – of the patient. If required, a new simulation should then guide the operator in setting the ventilator with respect to previous or modified goals. Obviously, many more studies will be needed before late outcomes of patients treated on the basis of goal-orientated ventilation can be evaluated.

Weaning from mechanical ventilation can be facilitated by computer algorithms based on measurements of a few physiological parameters and automatic control of the ventilator [31]. A computerized decision support system for ventilator management may significantly improve patient morbidity in ARDS [32]. These and other previous studies based on computer support aim to improve outcomes using a decision tree based on a few parameters. In the future, computer-supported ventilator setting may be derived from combinations of decision tree algorithms and simulations based on a detailed physiological platform, as in the present study.

In this study one goal was to maintain normocapnia, defined as Paco_2 _of 6 kPa. One could alternatively choose a higher value to follow the principle of permissive hypercapnia. The other goals were to minimise V_T _at a plateau pressure of 30 cmH_2_O. Taken together, the latter goals imply that PEEP_TOT _was maximized. Accordingly, the strategy was based on the concept that lung-protective ventilation is achieved at P_PLAT_below 30 cmH_2_O, and when repeated collapse and reopening of lung units is minimized by using small V_T_s and high PEEP. The validity of this concept was not examined in the present study. One could have chosen another strategy to demonstrate the feasibility of goal-orientated ventilation based on iterative computer simulation.

The animals were stabilised at a ventilator setting providing normocapnia at a P_PLAT _of 30 cmH_2_O at 40 breaths/min. In spite of the high starting frequency, further gains in terms of a modest but significant reduction in V_T _could be achieved at higher respiratory rates after one or two ventilator resettings based on computer simulation. Accordingly, goal-orientated ventilation by computer simulation is of potential clinical use even when ASPIDS is not applied. The optimal frequency indicated by computer simulation was about 64 breaths/min, which is much higher than usually applied in conventional mechanical ventilation. At RR_HIGH _and at ASPIDS, PEEP_TOT _was 18 and 17 cmH_2_O. This was efficient in maintaining lung aeration, as indicated by high Pao_2 _values. Also, in clinical ARDS an adequate PEEP value is efficient in this respect, even at low V_T _[33,34]. Obviously, dead space is the limiting factor for increasing RR. In mechanical ventilation, re-inspiration of carbon dioxide from the Y-piece and adjacent tubing contributes to dead space [35]. At RR_HIGH _this fraction was as high as 0.38. Another factor that contributes to dead space as a limiting factor for increased RR is that a shorter time for gas distribution and diffusion in the lungs leads to increased dead space [21,22]. By incorporating the concept of mean distribution time in the simulation, this factor was brought under control.

When ASPIDS was applied, V_T _could, after a single resetting based on computer simulation, be reduced to the very low value of 4 ml/kg, at 80 breaths/min on average. Measured Paco_2 _agreed with the value predicted by simulation. PEEP_TOT _and P_PLAT _were lower than predicted. Afterward, we determined that this was due to the reversal of flow in the tracheal tube during ASPIDS, which was not included in the simulation algorithm.

A RR of 80 breaths/min is often referred to the domain of high-frequency ventilation. However, fulfilment of goals at ASPIDS was achieved after simulation based mainly on classical parameters such as resistance, compliance, V_T _and dead space. An additional concept was that related to mean distribution time, which is particularly important at high respiratory rates.

Future experimental validation and development may be based on extended study periods comprising multiple resettings. Furthermore, data from routine ventilator resettings in ARDS patients may be used for validation. In this way, the system comprising data analysis of lung function and computer simulation can be validated without any risk to the patient. Another field of development is that of patient safety, but this may be carried out by ventilator manufacturers.

## Conclusion

By applying goal-orientated ventilation based on iterative computer simulation, V_T _could be reduced at high RRs and much more so by applying ASPIDS, while achieving the goals with respect to gas exchange and airway pressure. Classical physiological concepts complemented with that of mean distribution time are valid up to RRs between 60 and 80 breaths/min. Further studies of long-term effects of ASPIDS, guided by computer simulation, can pave the way for clinical studies in patients with critical lung disease.

## Key messages

• Goal-orientated mechanical ventilation is feasible, using a computer simulation based on a physiological profile.

• Aspiration of dead space allows an important reduction in V_T_, with sustained CO_2 _elimination.

• Studies of long-term effects of aspiration of dead space guided by computer simulation are needed.

## Abbreviations

ARDS = acute respiratory distress syndrome; ASPIDS = aspiration of dead space; Fio_2 _= fractional inspired oxygen; Paco_2 _= arterial carbon dioxide tension; Pao_2 _= arterial oxygen tension; PEEP = positive end-expiratory pressure; P_PLAT _= postinspiratory plateau pressure; RR = respiratory rate; V_E_co_2 _= expired volume of carbon dioxide; V_I_co_2 _= re-inspired volume of carbon dioxide; V_T_CO_2 _= eliminated tidal volume of carbon dioxide.

## Competing interests

The authors declare that they have no competing interests.

## Authors' contributions

LU participated in study design, data collection, data analysis and manuscript preparation. HÖ participated in data collection, data analysis and manuscript preparation. LN participated in data analysis and manuscript preparation. BD participated in software development and manuscript preparation. BJ participated in study design, data analysis and manuscript preparation. All authors read and approved the final manuscript.
